# Why transparency matters for sustainable data centers and carbon-neutral artificial intelligence (AI)

**DOI:** 10.1016/j.isci.2025.113705

**Published:** 2025-10-06

**Authors:** Can Hankendi, Ayse K. Coskun, Benjamin K. Sovacool

**Affiliations:** 1 Electrical and Computer Engineering Department, Boston University, Boston, MA, USA; 2 Department of Earth and Environment, Boston University, Boston, MA, USA

**Keywords:** Applied sciences, Computer science, Artificial intelligence

## Abstract

As artificial intelligence (AI) applications demand substantial computational power, their energy consumption and the attendant carbon footprint of data centers are accelerating at an alarming rate. Due to increasing demand, data centers could consume 9% of global electricity demand by 2030. However, the path toward more sustainable AI and carbon-neutral data centers is hindered by the lack of transparency in data sharing. Without access to operational data from data centers, researchers face limitations in developing effective solutions to minimize carbon emissions. Transparency is urgently needed to foster innovation, advance sustainability goals, and create practical strategies for reducing the environmental impact of AI and data centers. Transparency will help us to innovate around sustainability problems that are expected to become even harder to solve with the booming of the AI industry. Taking the necessary actions discussed in this article is essential to ensure a more sustainable future of the data center industry.

## Introduction

Artificial intelligence (AI) may be a boon for humanity, but a bane for the environment. Behind all the applications and services AI applications, algorithms, and programs promise to provide, physical—and often carbon intensive— infrastructures are needed to offer computing power, cloud storage, and material buildings behind AI architectures. This infrastructure, particularly data centers, is often carbon- and energy-intensive, posing challenges to sustainability. For instance, Google’s electricity usage just for AI operations is projected to exceed Google’s current data center total electricity consumption by 2034, highlighting the exponential growth in carbon emissions due to AI.^1^ Furthermore, globally, electricity consumption for AI operations is expected to reach to 800 TWh by 2026.^2^ Given recent trends, the carbon emissions of data centers pose significant risks to environmental sustainability.

The fundamental challenge arises from AI’s computational demands. Modern AI systems require massive computational resources both during training and inference phases. Training a single large language model can consume as much electricity as hundreds of U.S. homes use in a year, and these energy requirements continue to grow exponentially with model size and complexity.^2^ For example, Google’s electricity usage for AI operations alone is projected to exceed its current total data center electricity consumption by 2034.^1^ The International Energy Agency predicts global electricity consumption for AI operations will reach 800 TWh by 2026, equivalent to the annual electricity consumption of Japan.^2^

Despite growing awareness of these environmental challenges, a critical barrier prevents meaningful progress toward sustainable AI is the lack of transparency in data center operations. Technology companies typically treat operational data about energy usage, computational efficiency, and carbon emissions as proprietary information, limiting access to researchers and sustainability experts. This barrier to access to environmental data creates significant knowledge gaps about real-world AI energy consumption patterns and opportunities for optimization. Without comprehensive, accurate data from operational data centers, researchers are constrained in their ability to develop innovative solutions for reducing carbon emissions and improving energy efficiency in AI infrastructure.

The urgency of addressing this transparency gap has become even more pressing in recent years as AI applications have proliferated rapidly across industries. The emergence of generative AI systems has accelerated computational demands, which poses great environmental risks. Although the technical approaches to measure and mitigate AI’s carbon footprint have advanced, evaluating the effectiveness of these developments remains limited without access to comprehensive operational data.

In this Perspective, we examine how data transparency barriers currently prevent progress toward sustainable AI and carbon-neutral data centers. Our primary goals are to.

(1) Define the specific data gaps that hinder research and advancements in carbon-aware computing,
(2) Summarize and highlight global transparency efforts across regions and organizations,
(3) Propose a 10-year roadmap to guide academia, industry, and policymakers toward a more transparent and sustainable AI ecosystem.

Our main thesis is that achieving carbon-neutral AI is infeasible without first addressing transparency bottlenecks. Rather than presenting new experimental data, this Perspective synthesizes existing literature, sustainability reports, and public policy efforts to identify actionable steps forward. Although transparency alone cannot address all sustainability challenges in AI-driven carbon emissions, we believe improved data sharing can accelerate the development and implementation of necessary solutions.

### Unveiling the hidden sustainability costs of artificial intelligence and data centers

Today, data centers consume about 3% of the world’s electricity resources, which is expected to reach up to 9% by 2030.^3^ Given this trend, reducing carbon emissions, including embodied and operational footprints, and energy usage within data centers is essential for achieving sustainability goals set under the Paris Climate Accord, such as maintaining a 1.5-degree Celsius limit on worldwide temperature increases.

**Figure 1:**
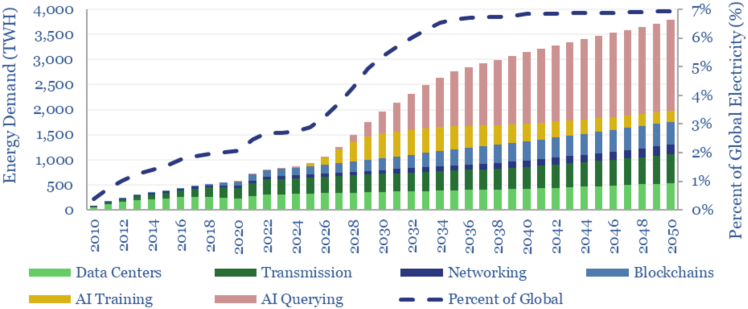
Breakdown of energy demand for various IT operations^5^

Recent developments in AI technologies have further exacerbated the sustainability challenges faced by data centers. Large AI models require significant computational power, which significantly increases the energy consumption and operational carbon footprint of data centers. For instance, training a mid-size AI model (i.e., 213M parameters) is estimated to generate as many carbon emissions as five cars over their lifetimes.^4^ As shown in Figure 1, the projected increase in AI-related energy demand could surpass that of conventional IT workloads by 2030. This makes the need for data transparency even more pressing. Without visibility into how resources are consumed, stakeholders cannot accurately assess where efficiency gains are possible or where regulatory intervention is most urgently needed.

Furthermore, as AI usage is becoming part of everyday activities, the carbon cost of AI inference is a major challenge to the environmental viability of data centers. A single AI-powered search query is estimated to consume 10 times more energy than a regular web search,^6^ highlighting the pressing need for new research directions to tackle the sustainability challenges posed by AI in the coming years. Recent studies such as Strubell et al.^4^ and Patterson et al.^7^ have quantified the power demands of large-scale model training, highlighting the vast environmental costs of modern AI. Industry leaders, including Google, Meta, and Amazon, now release high-level sustainability reports, disclosing aggregate Scope 1–3 emissions, renewable energy procurement, and data center efficiency metrics. However, these reports are insufficient for rigorous academic analysis. They typically lack granularity (e.g., per-inference energy use, real-time power draw), omit embodied carbon metrics for AI hardware, and do not follow standardized reporting formats. Chinnici et al.^8^ and De Vries^6^ emphasize the need for harmonized, verifiable emissions tracking, especially as AI operations become central to enterprise computing. Furthermore, major big-tech companies are committed to reducing their carbon emissions to net-zero by 2030. However, achieving these sustainability goals seems out of reach considering the increasing burden of AI technology on carbon emissions. Addressing the sustainability challenges requires research efforts and novel solutions.

The carbon footprint of data centers varies significantly across providers and regions. Microsoft’s data centers operate at a global average carbon intensity of 152 gCO_2_-eq/kWh, while Google reports 100 gCO_2_e/kWh and Amazon Web Services approximately 210 gCO_2_-eq/kWh according to their respective 2024 sustainability reports.^9^^,^^10^ These variations reflect differences in energy sourcing strategies, operational efficiency, and geographic distribution of facilities. Major technology companies have committed to achieving net-zero carbon emissions by 2030. However, recent projections suggest these goals will be difficult to achieve given AI’s growing energy demands. Google’s AI operations alone are projected to require 24–38 TWh of electricity by 2030, potentially increasing the company’s carbon footprint by 35–60% over current levels, assuming current energy sources remain unchanged.

Data are said to be the driving force of research, where accurate measurements and data availability are essential for developing novel solutions and advancing research. In the context of data centers and AI, these data include a wide range of metrics, from basic operational details to more complicated efficiency measures. However, most of the critical data points often remain inaccessible for researchers due to the industry’s reluctance to share information. Without access to thorough information on how data centers are built and operated, researchers rely on approximations and projections, which potentially lead to inaccurate conclusions.

Therefore, a lack of data transparency prevents further advancements in sustainable computing. By implementing transparency, it is possible to implement practical and accurate solutions to minimize the environmental impact of data centers. Designing and developing robust solutions requires diverse datasets across data centers, AI operations, and hardware manufacturing, which are mostly unavailable due to privacy and security concerns of private companies. We argue that data transparency is urgently needed to enable a greener digital future. This Perspective aims to highlight these gaps and present a collaborative path forward for data center developers, researchers, policymakers, and technology firms.

### Toward more transparent data

There are several reasons why data center operators and HW vendors are reluctant to publish detailed data on their operational and embodied carbon emissions. Data centers are considered mission-critical infrastructure; therefore, some operators are concerned that sharing too much information could put them at a disadvantage by becoming the focus of unwanted attention from cybercriminals and other bad actors. These security concerns prevent making any useful data available. Furthermore, private companies largely believe that disclosing certain information about their infrastructure, such as types of HW used, energy efficiency measures, might give away the competitive advantage.

Nevertheless, the quality of data related to data centers, including internal metrics such as server count, Power Usage Effectiveness (PUE), network architecture, and building specifications, is crucial for conducting comprehensive studies on the sustainability of data centers. For first order analysis carbon emission evaluation, it is possible to use the information regarding the area and power capacity of data centers from providers, while employing standard values for PUE, per-rack power, and average system utilization, as these specific data points are often not disclosed by data center operators. Commonly used metrics such as PUE, Carbon Usage Effectiveness (CUE), and Water Usage Effectiveness (WUE) provide standardized ways to evaluate data center resource efficiency.^11^ However, the implementation and reporting of these metrics remain inconsistent across the industry. The European Commission and several standardization organizations have emphasized the need for more consistent reporting standards to ensure comparability, transparency, and regulatory compliance.^12^^,^^13^

Furthermore, sustainability reports from companies such as Google, Meta, and Amazon provide some insights into their data center operations and environmental impact.^9^^,^^10^^,^^14^ These reports provide high-level data on operational/embodied carbon footprints (Scope 2–3), energy efficiency, renewable energy usage, and carbon reduction strategies. While this aggregate data are useful, it often combines multiple factors into a single metric, which can limit its utility for detailed research purposes.

We believe that enhancing data transparency, while ensuring the privacy of operators, is critical for facilitating future research aimed at addressing sustainability challenges associated with data center operations. In addition to sustainability reports, there are also both non-profit and for-profit efforts, such as Greenhouse Gas Protocol^15^ and Climatiq,^16^ to enable transparency on greenhouse gas emissions through providing access to historical emission databases for a multitude of industries, including IT and data center industries. Although these are significant initial steps toward data transparency, accessing recent and accurate data remains a significant challenge.

**Figure 2:**
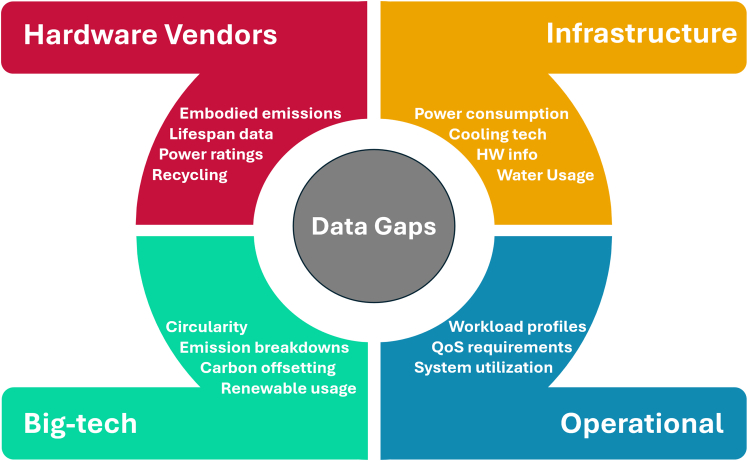
Categories of data gaps to enable sustainable AI and the data center industry

There are various types of data gaps that prevent the accurate evaluation of carbon emissions due to AI. Although there are efforts to make educated projections on AI carbon emissions, it remains challenging to put exact numbers and evaluate the actual impact of AI on the environment. Major data challenges are visualized in Figure 2, which categorizes data gaps that hinder progress in sustainable AI and data center operations. These include gaps that are multi-scalar and encompass operational data (e.g., power usage, real-time emissions), embodied emissions (e.g., hardware manufacturing and recycling), and external impacts (e.g., water usage, community effects). Each category illustrates a critical blind spot where data unavailability slows innovation and policy development.

Filling these data gaps requires a collaborative effort and responsible actions from all stakeholders. Without granular data, researchers are often left to rely on average models and estimates that can fail to accurately reflect the nuances of today’s increasingly large-scale hyperscale data center landscape. For instance, without having access to the actual PUE data or server utilization numbers, researchers are forced to resort to using industry average numbers, which often leads to over- or underestimating the actual carbon emissions.

On the other hand, the environmental impact of AI HW manufacturing, including GPUs, TPUs, and custom chips, is broadly unknown to researchers. Manufacturers of these HWs, such as NVIDIA, Google, choose not to release any information regarding the embodied footprint of manufacturing these devices. It is reported that only 60% of corporations disclose Scope 3 emissions, which account for more than 50% of total emissions.^17^ In addition to operational carbon footprint, quantifying the embodied footprint due to manufacturing, transportation, and recycling of HW devices designed for AI is essential to paint a full picture of the sustainability challenges due to AI. For instance, only 22% of electronic waste was recycled in 2022,^18^ and there are no standardized reporting methods to assess the impact of recycling on total emissions. Researchers are tackling these challenges by developing various architectural tools to estimate the embodied footprint of HW devices; however, reaching accurate models will require a significant amount of time and effort, which delays the urgent need for addressing the sustainability challenges.

Although some of these AI models are open-sourced for anyone to use, including researchers, the number of resources needed to train and evaluate these models is financially and practically very challenging for academia. Producing accurate research results and robust solutions is simply unviable given these circumstances. These data gaps prevent and slow down the pace of innovation in the data center industry, especially in AI, as the research on AI is significantly less established than CPU, networking, storage, and other areas of research in computing.

The social implications of the data center and AI industry are also largely difficult to assess. The impact of data centers on local communities, water usage, and land use needs to be studied to resolve any potential conflicts that it might lead to between data center operators and local communities. Designing data centers and developing ethical practices that benefit both the environment and society are crucial for a sustainable future.

Despite these hurdles, there are continued efforts to increase transparency in the industry. Initiatives such as the Open Compute Project (OCP)^19^ and The Green Grid,^20^ researchers hope to standardize carbon emission reporting to enable transparency. The OCP, for example, aims to make both design and operational data available to researchers to enable new research directions. However, these initiatives are still in their early stages and have not been widely adopted in the research community.

### Regional approaches and case studies

The organizations in Europe and Japan have established comprehensive frameworks to ensure data transparency and ethical data usage in their high-performance computing (HPC) and research initiatives. These frameworks are designed to establish trust, accountability, and innovation while addressing the growing demands for secure and transparent data handling in scientific and industrial applications.

In Europe, the EuroHPC Joint Undertaking (JU) plays a crucial role in creating a federated supercomputing ecosystem that emphasizes accessibility, transparency, and ethical data practices. EuroHPC’s mission is to provide HPC resources to a wide range of users, including public institutions, private companies, and researchers, while ensuring that data usage aligns with ethical and transparent standards. To maintain transparency in decision-making, EuroHPC relies on its Industrial and Scientific Advisory Board, which provides independent guidance on strategic research and innovation priorities.

The Gauss Alliance in Germany complements EuroHPC’s efforts by supporting the sustainable and efficient use of supercomputing resources. The alliance focuses on transparent data management and accessibility, ensuring that scientific and industrial users can leverage HPC resources while adhering to national and European data standards. It collaborates with initiatives such as the National Grid Initiative for Germany (NGI-DE), which promotes transparent data sharing. This collaborative approach ensures that data usage policies are consistent and aligned with broader European sustainability goals.

Similarly, the National High-Performance Computing (NHR) centers in Germany operate under strict transparency and accountability frameworks. Funded by federal and state governments, these centers undergo rigorous scientific evaluation by the German Research Foundation (DFG) to ensure efficient and transparent resource allocation. The NHR centers also support cutting-edge research in AI and data-intensive applications, emphasizing ethical data usage and the transparent dissemination of research outcomes. By fostering a culture of accountability, the NHR centers contribute to the broader European vision of a transparent and accessible HPC ecosystem.

In Japan, RIKEN has implemented advanced systems and policies to ensure data transparency and security in its research activities. One notable initiative is the HOKUSAI-SR system, a secure analysis environment designed to protect personal data while enabling transparent and ethical research practices. This system simplifies compliance with Japan’s strict personal information protection laws, bioethics regulations, and information security standards, ensuring that data usage is both transparent and secure. RIKEN’s Information Security Policy further reinforces this commitment by providing clear guidelines for data protection and regularly updating its regulations to reflect evolving legal and ethical requirements.

In North America, transparency initiatives have emerged in both industry and academic-industry partnerships. The Open Compute Project (OCP), which was initiated by Meta, has significantly advanced hardware design transparency by publishing detailed specifications for energy-efficient server designs.^19^ Microsoft’s Project Natick is another notable case study, which provides transparency into underwater data center operations.^21^ The project published comprehensive datasets on cooling efficiency, server reliability, and power consumption patterns under varying oceanic conditions. Analysis of this transparent data showed that underwater deployments achieved an average PUE of 1.07 compared to 1.25 for conventional data centers.^21^

Singapore’s Green Data Center Innovation Program represents a government-led approach to transparency, requiring data centers to disclose energy efficiency metrics and incentivizing improvements through tax benefits.^22^ However, power data, especially from Asian economies still hard to get access to. It is reported that more than half of Asian economies fail to provide reliable power data.^23^

These diverse regional approaches demonstrate that transparency initiatives are evolving globally. They also show that greater transparency in computing infrastructure operations leads to substantial efficiency improvements and enhanced innovation in sustainability solutions.

### Challenges in data transparency

The path toward improved data transparency in AI and data center operations faces significant obstacles spanning technical, commercial, and regulatory domains. A primary obstacle to data transparency is the fact that operational data constitutes proprietary information critical to competitive advantage. Major cloud providers and data center operators have invested billions in optimizing their infrastructure, and many consider their energy efficiency techniques, cooling strategies, and workload management approaches to be trade secrets. This approach causes an issue between transparency and business interests. For instance, when researchers requested detailed power consumption data from ten major data center operators for a comprehensive efficiency study in 2019, only two provided partial data, with the remainder citing competitive concerns.^24^

Transparency efforts are further complicated by the technical complexity of modern data centers and the lack of standardized measurement methodologies. Various metrics exist for assessing data center efficiency (e.g., PUE, CUE, WUE, and so forth), but implementation and reporting methodologies vary widely, which makes accurate comparisons difficult. A 2023 study of 122 data centers found that PUE calculations varied by up to 0.4 points (approximately 20% difference) due to inconsistent measurement methodologies.^13^

The emergence of AI workloads has introduced additional complexity, as traditional efficiency metrics fail to capture the relationship between computational needs and energy consumption adequately. Standard benchmark tests such as SPECpower, measure general computing efficiency but do not address specialized AI workloads. This gap has led to inconsistent reporting and difficulty in assessing the true environmental impact of AI operations.

On the other hand, detailed operational data can inadvertently reveal sensitive information about infrastructure configurations, security protocols, and business operations. For instance, granular power consumption data might expose usage patterns that indicate customer activity levels or reveal when security systems are operating at reduced capacity. These security concerns create tension between transparency goals and privacy concerns.

### Roadmap for future developments

**Figure 3:**
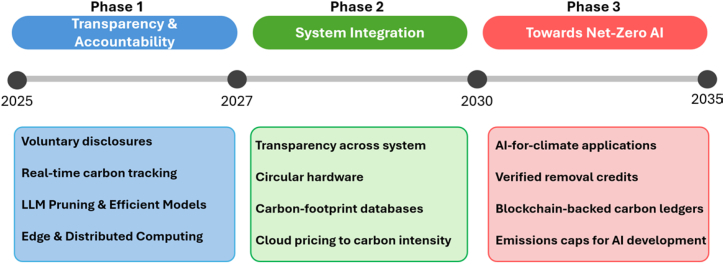
10-year roadmap of actionable items to achieve net-zero AI

To mitigate the environmental costs of artificial intelligence, a systematic approach to data transparency is essential. This section outlines a three-phase roadmap (2025–2035) to align AI development with climate goals, addressing transparency, accountability gaps, and system-wide changes, as illustrated in Figure 3. Each phase requires collaboration with technical and regulatory actions to ensure measurable progress.

#### Phase 1: Transparency and accountability (2025–2027)

The initial phase focuses on establishing baseline transparency mechanisms to quantify and mitigate AI’s environmental impact. Governments must take the led by expanding regulatory frameworks such as the EU AI Act to mandate comprehensive Scope 1–3 emissions reporting for AI infrastructure, including cloud providers and hardware manufacturers. At the same time, the research community should adopt standardized measurements such as Hugging Face’s Energy Score to benchmark energy use per model inference to ensure that the publications include carbon cost alongside performance metrics.^25^ Alongside policy changes, large cloud providers and data center operators should be encouraged to participate in pilot programs for voluntary disclosure, particularly in collaboration with academic and nonprofit researchers. These efforts can be modeled after frameworks from other sectors, such as the Global Alliance for Genomics and Health (GA4GH)^26^ in healthcare or the Financial Data Exchange (FDX)^27^ in finance, that have successfully fostered data sharing through voluntary participation and governance. On the technical front, prioritizing sparse neural architectures and quantization techniques can further help reduce training energy demands. Key initiatives in this phase include voluntary disclosures of real-time carbon emissions to enable stakeholders to track environmental impacts dynamically. To reduce energy demand, this phase emphasizes technical optimizations such as Large Language Model (LLM) pruning, a technique that removes redundant parameters without compromising performance, and the adoption of energy-efficient architectures. Further, a shift toward edge and distributed computing reduces reliance on centralized data centers, minimizing transmission losses and improving scalability.

#### Phase 2: System integration (2027–2030)

The second phase targets the system-wide integration of sustainability across AI’s life cycle and supply chains. One critical step is transitioning to circular hardware economies, where GPU recycling programs and low-carbon alternatives could reduce embodied emissions. Policy efforts such as the EU’s Right-to-Repair Act can extend hardware lifespans, while cloud platforms might implement carbon-aware pricing models that penalize fossil-fueled compute during peak grid demand. Furthermore, collaborative efforts will be essential to develop sustainability-oriented open benchmarks to track end-to-end emissions from data centers to end-user devices. Concurrently, this phase enforces policy-driven actions such as emissions caps for AI training and deployment, ensuring that growth aligns with sustainability goals.

Furthermore, carbon-aware pricing models should be introduced to disincentivize fossil-fueled computation, particularly during periods of grid stress. These could be enforcing higher electricity rates for energy-intensive AI training jobs run on carbon-intensive grids or time-of-use pricing that favors cleaner energy windows. However, such penalties must be backed by enforceable policy mechanisms and aligned with real-time electricity market signals. Without proper regulatory oversight, the monetary benefits of using fossil fuels may outweigh the sustainability-related penalties, making them ineffective. A coordinated approach that combines penalties with caps, incentives, and transparent reporting will be essential.

#### Phase 3: Toward net-zero artificial intelligence (2030–2035)

The final phase aims for net-zero AI through innovation and strict standards. AI’s capabilities can be utilized toward climate solutions, such as optimizing carbon capture. This phase relies on innovation in renewable energy adoption, advancements in low-power hardware, and reliable carbon removal technologies. Furthermore, it highlights the need for cross-domain collaboration, policy alignment, and open research to address challenges in measuring and mitigating AI’s environmental footprint. To mitigate excessive resource use, emissions budgets (e.g., capped at 100 tons CO_2_eq per training run) could be enforced. To ensure verifiability, blockchain-backed carbon records can be utilized to provide immutable records of emissions data and mitigation efforts.

By prioritizing transparency, integrating carbon-aware systems, and committing to net-zero targets, the AI industry can mitigate its environmental impact while maintaining technological progress. By promoting accountability from detailed emissions tracking to hardware circularity, the AI infrastructure can sustainably continue its exponential growth that aligns with environmental goals. Policymakers, researchers, and corporations must collaborate to translate these phases into actionable progress.

## Conclusion

Implementing data transparency practices would benefit not only stakeholders in the data center industry, including researchers, operators, and users, but society at large in terms of achieving energy, climate, and sustainability objectives. Enabling researchers to access detailed data would improve the quality and the impact of the sustainability research. On the other hand, operators would benefit from research output and improve their efficiency to achieve their sustainability goals. It could also foster a more collaborative industry culture, where companies work together to solve common challenges.

There are data consortia and public-private partnerships established in various industries, including health,^26^ finance^27^ and IT,^28^ to facilitate data sharing across stakeholders while maintaining privacy. Similarly, there are sustainability focused data transparency initiatives, such as *Global Reporting Initiative*^29^ and *The Open Data Institute*^30^ that are tackling the data privacy, data sharing, and regulatory compliance issues. Applying these models to the AI and data center industry could standardize and accelerate data transparency efforts.

For policymakers and the public, transparency is essential for accountability. Access to transparent data would also enable regulators to keep companies in line with environmental standards. Finally, transparency will enable policymakers to implement data-driven public policies that are not based on assumptions and incomplete information.

There is no straight path to greater transparency, but it is also a prerequisite for a more sustainable data center industry. Collectively, industry leaders and researchers, in collaboration with policymakers, need to set the required standards that incentivize or regulate transparency so that the industry’s growth does not come at the expense of its accountability, legitimacy, and sound governance.

## Acknowledgments

This project is funded 10.13039/501100005416Research Council of Norway (Award #344115).

## Author contributions

Writing, editing, original draft, investigation, and methodology, C.H.; conceptualization, supervision, and funding acquisition, A.K.C. and B.K.S.

## Declaration of interests

The authors declare no competing interests.

## References

[1] Castro D. (2024). Rethinking concerns about AI’s energy use. Center For Data Innovation.

[2] IEA (2024). https://iea.blob.core.windows.net/assets/18f3ed24-4b26-4c83-a3d2-8a1be51c8cc8/Electricity2024-Analysisandforecastto2026.pdf.

[3] EPRI (2024). https://www.epri.com/research/products/3002028905.

[4] Strubell E., Ganesh A., McCallum A., McCallum A. (2020). Energy and policy considerations for modern deep learning research. Proc. AAAI Conf. Artif. Intell..

[5] Thundersaid Energy (2024). Internet energy consumption: Data, models, and forecasts. https://thundersaidenergy.com/downloads/internet-energy-consumpion-data-models-forecasts/.

[6] de Vries A. (2023). The growing energy footprint of artificial intelligence. Joule.

[7] Patterson D., Gonzalez J., Le Q.V., Liang C., Munguia L.M., Rothchild D., So D.R., Texier M., Dean J. (2021). Carbon Emissions and Large Neural Network Training. arXiv.

[8] Chinnici M., Mores R., Riccardi R., Vitale G. (2022). Carbon-aware data center scheduling: challenges and opportunities. Sustainable Computing: Informatics and Systems.

[9] Google (2024). Google 2024 environmental report. https://sustainability.google/reports/google-2024-environmental-report/.

[10] Amazon (2023). 2023 sustainability report. https://sustainability.aboutamazon.com/2023-sustainability-report.pdf.

[11] Belady C., Rawson A., Pfleuger J., Cader T. (2008).

[12] European Commission (2021). https://data.europa.eu/doi/10.2759/912563.

[13] Avgerinou M. (2023). Global trends, performance metrics, and energy reduction measures in datacom facilities. Renew. Sustain. Energy Rev..

[14] Meta (2024). Meta 2024 sustainability report. https://sustainability.atmeta.com/wp-content/uploads/2024/08/Meta-2024-Sustainability-Report.pdf.

[15] GHG Protocol (2024). Greenhouse gas protocol. https://ghgprotocol.org/.

[16] Climatiq (2024). Climatiq: Sustainability and carbon footprint management. https://www.climatiq.io/.

[17] (Website) Clarity AI (2024). *Carbon reporting trends: Has global progress stalled?* Clarity AI. https://clarity.ai/research-and-insights/climate/carbon-reporting-trends-has-global-progress-stalled/.

[18] WHO (2024). Electronic waste (e-waste). https://www.who.int/news-room/fact-sheets/detail/electronic-waste-(e-waste.

[19] OCP (2024). Open Compute Project: *Sustainability project overview*. Open Compute Project.

[20] The Green Grid (2024). The Green Grid: Advancing resource efficiency in IT and data centers. The Green Grid. https://www.thegreengrid.org/.

[21] Microsoft (2020). *Project Natick: Microsoft's Underwater Data Center*. Microsoft. https://www.microsoft.com/en-us/research/project/project-natick/.

[22] IMDA (2024). Charting green growth pathways at scale for data centres in Singapore. https://www.imda.gov.sg/resources/press-releases-factsheets-and-speeches/factsheets/2024/charting-green-growth-for-data-centres-in-sg.

[23] Ember (2023). Asia data transparency report 2023. https://ember-energy.org/latest-insights/asia-data-transparency-report-2023/.

[24] Masanet E. (2020). Recalibrating global data center energy-use estimates. Science.

[25] Hugging Face (2024). *Announcing AI Energy Score ratings*. Hugging Face Blog. https://huggingface.co/blog/sasha/announcing-ai-energy-score.

[26] GA4GH (2024). Global Alliance for Genomics and Health (GA4GH). https://www.ga4gh.org/.

[27] FDX (2024). Financial Data Exchange (FDX). https://financialdataexchange.org/.

[28] GAIA-X. (2024). GAIA-X: A Federated Data Infrastructure for Europe. https://gaia-x.eu/.

[29] GRI (2024). Global Reporting Initiative (GRI). https://www.globalreporting.org/.

[30] ODI (2024). The Open Data Institute (ODI). https://theodi.org/.

